# Colonic bleeding from migrated cement

**DOI:** 10.1002/ccr3.1511

**Published:** 2018-04-10

**Authors:** Georgios C. Sotiropoulos, Zoe Garoufalia, Christos Klonaris, Nikolaos Machairas

**Affiliations:** ^1^ 2nd Department of Propaedeutic Surgery National and Kapodistrian University of Athens, Medical School Athens Greece

**Keywords:** Cement, colon, foreign body, migration

## Abstract

Migration of foreign bodies through the colonic wall can be the result of inflammation or effect of radiotherapy. Patients with such condition may remain asymptomatic. Careful assessment of patient' history can provide valuable information in symptomatic cases such as colonic obstruction or bleeding.

## Case Presentation

**Figure 1:**
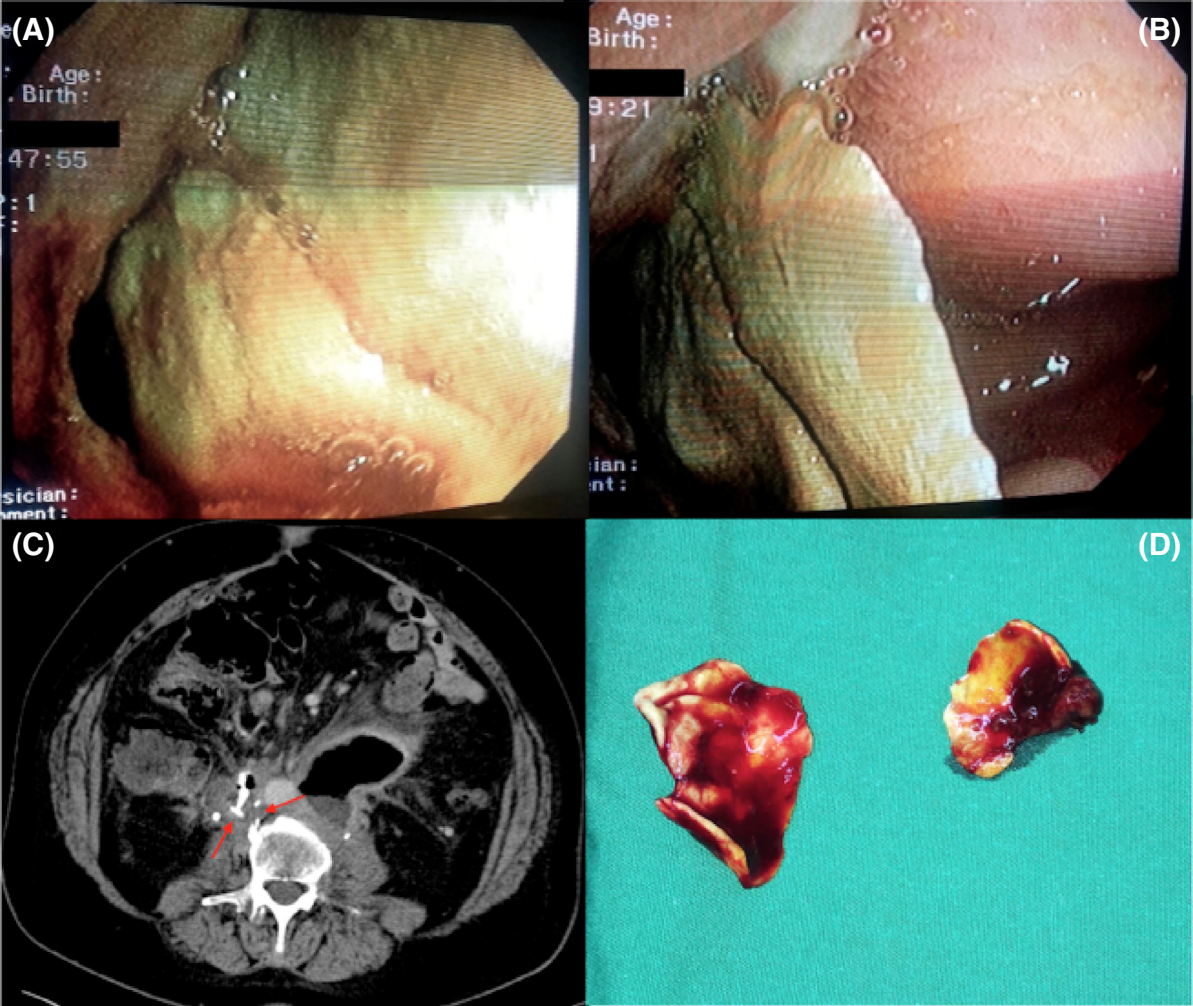
(A–B) Colonoscopy images of the foreign body invading through the colonic wall, (C) Computer tomography image of the detached cement, (D) Fragments from the foreign body after resection.

A 63‐year‐old male patient was urgently admitted to our hospital for hematochezia. The patient had a history of multiple resections and radiotherapy sessions for recurrent retroperitoneal liposarcoma. He underwent urgent colonoscopy, which revealed a solid, partially obstructing foreign body in the ascending colon, which derived from the colonic wall (Fig. 1A and B). Computer tomography (CT) was initially inconclusive concerning the foreign body origin. The patient received coagulative agents and fresh frozen plasma and the bleeding stopped.

As imaging studies were indecisive in establishing a diagnosis, the patient's history was reassessed. In one operation 50 months prior to presentation of current symptomatology, part of the abdominal aorta and the vena cava were removed en‐block with the tumor, the vena cava was ligated, and aortic reconstruction using a Y‐shaped graft was performed. Additionally, orthopedic cement was applied on the retroperitoneal surface paraspinal as a salvage solution due to lumbar veins plexus diffuse bleeding. CT revision confirmed the intraluminal presence in the colon ascendens of the piece of cement, which had progressively migrated, eroded, and infiltrated the colic wall (Fig. 1C). After optimization, the patient underwent explorative laparotomy with right colectomy and removal of the foreign body (Fig. 1D).

## Conflict of Interest

None declared.

## Authorship

GCS and NM: designed and conceived the study. GCS and CK: acquired the data. NM and ZG: wrote the paper. GCS and NM: analyzed and interpreted the data.

